# Automated Segmentation of Fluorescence Microscopy Images for 3D Cell Detection in human-derived Cardiospheres

**DOI:** 10.1038/s41598-019-43137-2

**Published:** 2019-04-30

**Authors:** Massimo Salvi, Umberto Morbiducci, Francesco Amadeo, Rosaria Santoro, Francesco Angelini, Isotta Chimenti, Diana Massai, Elisa Messina, Alessandro Giacomello, Maurizio Pesce, Filippo Molinari

**Affiliations:** 1 Department of Electronics and Telecommunications, Politecnico di Torino, Turin, 10129 Italy; 2 Department of Mechanical and Aerospace Engineering, Politecnico di Torino, Turin, 10129 Italy; 3 Unità di Ingegneria Tissutale Cardiovascolare, Centro Cardiologico Monzino, IRCSS, Milan, 20138 Italy; 4 Department of Medical-Surgical-Sciences and Biotechnology, Sapienza University of Rome, Rome, 00185 Italy; 5 Mediterranea, Cardiocentro, Napoli, 80122 Italy; 6 Department of Pediatrics and Neuropsychiatry, “Umberto I” Hospital, Rome, 00161 Italy; 7 Department of Molecular Medicine, Sapienza University of Rome, Rome, 00185 Italy

**Keywords:** Fluorescence imaging, Image processing, Heart stem cells

## Abstract

The ‘cardiosphere’ is a 3D cluster of cardiac progenitor cells recapitulating a stem cell niche-like microenvironment with a potential for disease and regeneration modelling of the failing human myocardium. In this multicellular 3D context, it is extremely important to decrypt the spatial distribution of cell markers for dissecting the evolution of cellular phenotypes by direct quantification of fluorescent signals in confocal microscopy. In this study, we present a fully automated method, named CARE (‘CARdiosphere Evaluation’), for the segmentation of membranes and cell nuclei in human-derived cardiospheres. The proposed method is tested on twenty 3D-stacks of cardiospheres, for a total of 1160 images. Automatic results are compared with manual annotations and two open-source software designed for fluorescence microscopy. CARE performance was excellent in cardiospheres membrane segmentation and, in cell nuclei detection, the algorithm achieved the same performance as two expert operators. To the best of our knowledge, CARE is the first fully automated algorithm for segmentation inside *in vitro* 3D cell spheroids, including cardiospheres. The proposed approach will provide, in the future, automated quantitative analysis of markers distribution within the cardiac niche-like environment, enabling predictive associations between cell mechanical stresses and dynamic phenotypic changes.

## Introduction

Monitoring the differentiation process of stem/progenitor cells is important either to devise new regenerative medicine approaches, or to understand the molecular basis of chronic diseases involving modifications in tissue structure and property^1^. Until now, this issue has remained relatively unaddressed, also given the lack of systematic tools enabling quantitative investigation (even in real time) of cells dynamics inside the so-called stem cell niches^2^ or in disease models^3,4^. In the last decade the need for quantitative, cost-effective methods for analyzing, e.g., cell-matrix as well as cell-cell dynamic interactions, has become more and more compelling. The need for quantitative tools is being stimulated by the plethora of methods recently proposed to engineer tissue-specific 3D microenvironments mimicking the native architecture, i.e. the so-called ‘organoid’ approach^5^. This approach is expected in the future to support ‘synthetic’ tissue/niche modelling^6^ for enhanced regenerative medicine applications^7^, pathology decryption^8^ or fundamental cell differentiation programs in developmental processes^9,10^.

In this context, the cardiosphere is a representative model of cardiac niche, which may be suitable for myocardial regeneration/engineering approaches^11–13^, as well for decryption and modelling of molecular mechanisms underlying myocardial diseases, such as, for example, cardiac fibrosis^14,15^. Cardiospheres are cultured taking advantage of the natural ability of stromal progenitors to (1) outgrow from explanted human adult-derived atrial appendage tissue, and (2) aggregate onto cell-repulsive culture substrates^16^, thereby maintaining cell to cell contacts. Previous studies have already demonstrated that the outer and the inner cardiosphere environments present remarkable differences, as far as differentiation potency, paracrine signaling and metabolism are concerned (a detailed discussion can be found, e.g., in ref.^15^). Up to now, only approaches with limited quantitative throughput have been applied to assess cell morphology and dynamics inside these complex structures, mostly based on fluorescence-based imaging technology^17^.

Cell segmentation within fluorescence microscopy images is a challenging task for an automated algorithm. First of all, the autofluorescence from out of focus tissues causes an irregular background intensity. This irregularity makes the distinction of the foreground from the background a challenging task. Moreover, the variation of nuclei intensity within the same image also complicates the automatic cell separation, causing over-segmentation during the nuclei detection^18^. Most current nuclei detection approaches in fluorescence microscopy images are based on intensity thresholding^19^ and gradients^20^. However, all of these methods have been developed to analyze 2D images and none of these has been applied in a multicellular 3D context.

To bridge the gap of knowledge derived by a paucity of automatic solutions for the specific characterization of cells inside *in vitro* 3D aggregates, including cardiospheres, here an adaptive algorithm is presented, CARE (‘CARdiosphere Evaluation’), for automatic cardiosphere segmentation in fluorescence microscopy images. The proposed technique takes a 3D stacked image from confocal microscopy as input and performs the segmentation and 3D rendering of cardiosphere membranes and nuclei. The CARE algorithm was tested on 1160 fluorescent images of human-derived cardiospheres. Manual annotations were compared with automatic results provided by CARE and two open-source software designed for cell detection (Fiji and CellProfiler).

## Results

### Primary derivation and confocal microscopy analysis of human cardiospheres

**Figure 1:**
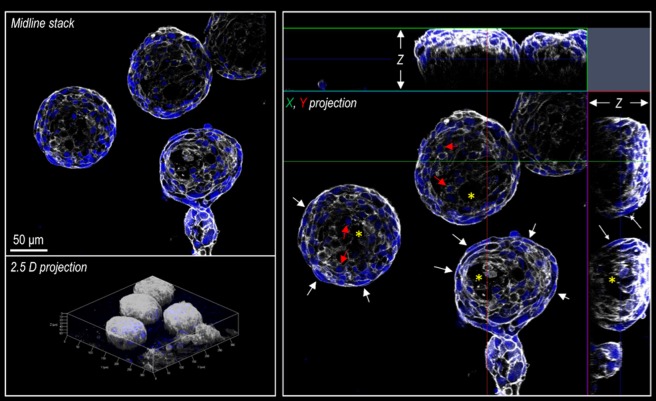
Structure of the cardiospheres as observed by confocal microscopy. White color represents the cytoskeleton as evidenced by F-actin staining with Phalloidin. Blue color represents the nuclei as revealed by DAPI, an intercalant of the DNA. The three panels represent the midline stack (upper left) the 2.5 projection of the cardiospheres with the X and Y dimensions (lower left) and the projection of the whole stack along the indicated X and Y axes (right) respectively.

Cardiospheres were stained with DAPI, and TRITC-labelled phalloidin to highlight, respectively, cells and nuclei distribution and shapes^16^. Conventional confocal microscopy was employed to obtain images of the cardiospheres by 3D-stack acquisition with a relatively high definition. By visual inspection of Fig. 1, cardiospheres exhibited a complex structure emerging above the culture plate as hemispheres, made of cells distributed with apparent multiple orientation and cells/nuclei shapes. The presence of internal cavities with a non-uniform dimension can be also appreciated.

### CARE vs. manual operator image segmentation

All the 1160 images of the dataset are used to validate the performance of CARE in segmenting cardiospheres borders respect to two manual operators (OP1, OP2). Given the presence of a high number of nuclei in each image, only part of them was used to validate the DAPI layer. In particular, five random images are extracted from each stack, for a total of 100 images. The same two operators manually draw each cell in order to assess inter-operator variability in the cell nuclei detection.

A comparison between masks drawn by a manual operator (MASK_MANUAL_) and those provided by CARE (MASK_AUTOMATIC_) is also carried out to assess the algorithm performance in the segmentation of cardiosphere borders and cell nuclei. The segmentation performance was calculated using the *recall*, *precision*, *F1*_*SCORE*_ and *jaccard*_*INDEX*_, defined as follows:

1 $$recall=\frac{TP}{TP+FN}$$

2 $$precision=\frac{TP}{TP+FP}$$

3 $$F{1}_{SCORE}=\frac{2\times (recall\times precision)}{recall+precision}$$

4 $$jaccar{d}_{INDEX}=\frac{|MAS{K}_{MANUAL}\cap MAS{K}_{AUTOMATIC}\,|}{|MAS{K}_{MANUAL}\cup MAS{K}_{AUTOMATIC}|}$$

True positive (TP) denotes the number of pixels in common between manual and automatic masks, false negative (FN) represents all pixels not identified by CARE and false positive (FP) are all the pixels identified by CARE but not by the manual operator. In detail, *recall* measures the missed detection of ground truth shapes, *precision* evaluates the false detection of ghost objects, *F1*_*SCORE*_ is defined as the harmonic mean of *recall* and *precision*^21^, and the *jaccard*_*INDEX*_ measures similarity between two different shapes, defined as the size of the intersection divided by the size of the union of the segmented object^22^.

The results of the comparison between manual and automatic segmentation are summarized in Table 1. CARE demonstrated excellent performances in segmenting cardiospheres borders (PHAL), with very high average values of *precision*, *recall*, *F1*_*SCORE*_ and *jaccard*_*INDEX*_ respect to two expert operators (OP1, OP2) thus demonstrating the accuracy of the method (Table 1). As for nuclei segmentation (DAPI), the average *F1*_*SCORE*_ calculated between the two operators (0.7872) is comparable with the one obtained between CARE and each of them (0.7679 and 0.7615). The algorithm exhibited an excellent performance in the recognition of cell nuclei, compared to manual operators (OP1: 0.9001 and OP2: 0.9210). Being very sensitive, CARE tends to slightly overestimate the nuclei surface, and this leads to a lower *precision*, compared to manual operators (OP1: 0.6713 and OP2: 0.6517). Moreover, no statistical difference was found in the precision and recall values in the inner and outer environment of the cardiosphere, thus demonstrating the efficiency of the proposed nuclei detection (Table 2). The values of *jaccard*_*INDEX*_, between OP1 and CARE (0.6157), and OP2 and CARE (0.6174) were observed to be comparable to the value between OP1 and OP2 (0.6497).

**Table 1:** Performance of the proposed method in the cardiosphere border (PHAL) and nuclei segmentation (DAPI).

Layer	#Images	Validation	Recall	Precision	F1_SCORE_	jaccard_INDEX_
PHAL	1160	OP1 vs OP2	0.9410 ± 0.0285	0.9789 ± 0.343	0.9588 ± 0.0158	0.9170 ± 0.0281
		OP1 vs CARE	0.9339 ± 0.0241	0.9728 ± 0.0345	0.9497 ± 0.0157	0.9079 ± 0.0255
		OP2 vs CARE	0.9508 ± 0.0264	0.9717 ± 0.0351	0.9606 ± 0.0201	0.9271 ± 0.0255
DAPI	100	OP1 vs OP2	0.7602 ± 0.0486	0.8215 ± 0.0507	0.7872 ± 0.0234	0.6497 ± 0.0318
		OP1 vs CARE	0.9001 ± 0.0329	0.6713 ± 0.0506	0.7679 ± 0.0370	0.6157 ± 0.0431
		OP2 vs CARE	0.9210 ± 0.0316	0.6517 ± 0.0599	0.7615 ± 0.0439	0.6174 ± 0.0586

Data are reported as mean ± standard deviation.

**Table 2:** Comparison between the segmentation performance in the external shell (precision_OUTER_, recall_OUTER_) and the one obtained in the internal ‘core’ of the cardiosphere (precision_INNER_, recall_INNER_).

Layer	#Images	Validation	Precision_INNER_	Precision_OUTER_	Recall_INNER_	Recall_OUTER_
DAPI	100	OP1 vs CARE	0.6735 ± 0.1342	0.6584 ± 0.1341	0.8817 ± 0.0980	0.8760 ± 0.0720
		OP2 vs CARE	0.6510 ± 0.1510	0.6472 ± 0.1484	0.9074 ± 0.0659	0.8966 ± 0.0671

**Figure 2:**
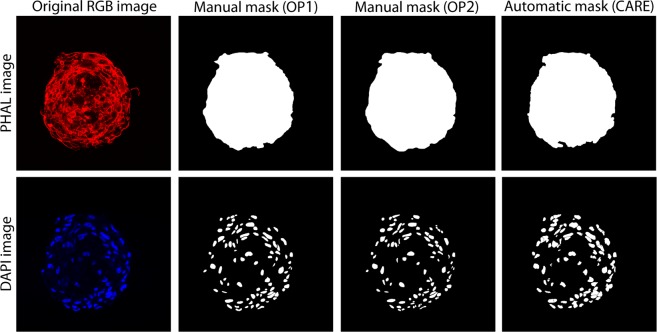
Comparison between manual and automatic segmentation (rows). First column shows the original RGB image while manual masks performed by two expert operators (OP1, OP2) are reported in the second and third columns. The result provided by the proposed method is shown in the rightmost column.

Finally, a Kruskal-Wallis test^23^ is used to compare the inter-operator variability (OP1 vs OP2) with the automatic performance (OP1 vs CARE, OP2 vs CARE). The Kruskal-Wallis test works under the null-hypothesis that the data comes from the same distribution (p-value was set to 0.05). For both PHAL and DAPI layer, the Kruskal-Wallis test confirmed that there was no statistical difference between inter-operator variability (OP1 vs OP2) and automatic performance (OP1 vs CARE, OP2 vs CARE) for *F1*_*SCORE*_ and *jaccard*_*INDEX*_ distributions (p-value > 0.05). We also conducted an analysis of the number of nuclei identified by the 2 operators (OP1 and OP2) and CARE. No statistical difference was found between manual and automatic cell counting (Table 3). An explanatory example comparing the output of the segmentation obtained by applying CARE and by manual operators is presented in Fig. 2.

**Table 3:** Comparison between manual (#Nuclei OP1, #Nuclei OP2) and automatic (#Nuclei CARE) cell counting.

Layer	#Images	#Nuclei OP1	#Nuclei OP2	#Nuclei CARE
DAPI	100	81.01 ± 46.24	79.42 ± 45.81	80.84 ± 42.30

### Comparison with open-source softwares

**Figure 3:**
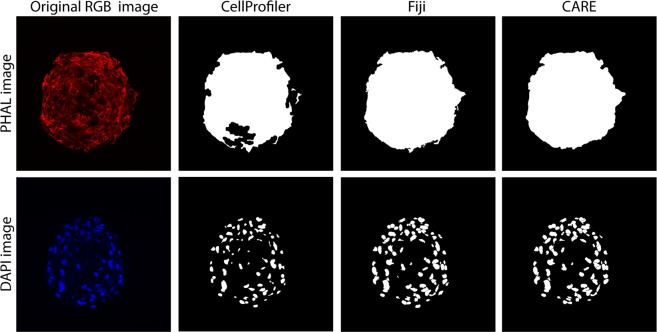
Comparison between two open-source software and the proposed method in the cardiosphere segmentation (rows). First column shows the original RGB image while CellProfiler^23^ and Fiji^24^ result are provided in the second and third columns respectively. The automatic mask obtained with CARE is shown in the last column.

The CARE automatic segmentation was also compared with two open-source softwares (CellProfiler and Fiji) widely applied to the analysis of fluorescence microscopy images. CellProfiler^24^ is composed of a series of image-processing modules that allow the user to perform an automatic analysis. Fiji^25^ is a Java-based software with several plugins which facilitate scientific image analysis based on a semi-automatic pipeline consisting of: (i) conversion of RGB image into grayscale, (ii) manual intensity thresholding, (iii) hole filling and (iv) small particles removal. For the nuclei segmentation, here an additional step was included in the analysis: (v) automatic cell separation. A visual inspection of Fig. 3 allows to compare the performances of CellProfiler, Fiji and CARE in cardiospheres segmentation. A quantitative comparison of the performances offered by the two open-source software with CARE is reported in Tables 4 and 5.

**Table 4:** Performance of two open-source software (CellProfiler, Fiji) in the segmentation of the external cardiosphere membrane (PHAL layer). Data are reported as mean ± standard deviation.

Method	Computational Time (sec)	PHAL layer			
		Recall	Precision	F1_SCORE_	jaccard_INDEX_
CellProfiler (automatic)	13.18 ± 4.51	0.7997 ± 0.0655	0.9912 ± 0.0028	0.8724 ± 0.0482	0.7939 ± 0.0671
Fiji (semi-automatic)	117.21 ± 13.91	0.8865 ± 0.0559	0.9902 ± 0.0005	0.9249 ± 0.0387	0.8775 ± 0.0548
**CARE (proposed)**	**8**.**27 ± 1**.**31**	**0**.**9339 ± 0**.**0241**	**0**.**9728 ± 0**.**0345**	**0**.**9497 ± 0**.**0157**	**0**.**9079 ± 0**.**0255**

**Table 5:** Performance of two open-source software (CellProfiler, Fiji) in the segmentation of the cardiosphere cell nuclei (DAPI layer). Data are reported as mean ± standard deviation.

Method	Computational Time (sec)	DAPI layer			
		Recall	Precision	F1_SCORE_	jaccard_INDEX_
CellProfiler (automatic)	14.26 ± 3.02	0.8053 ± 0.1198	0.5818 ± 0.1704	0.6536 ± 0.1341	0.5031 ± 0.1365
Fiji (semi-automatic)	123.81 ± 19.38	0.8457 ± 0.0944	0.6845 ± 0.0453	0.7504 ± 0.0522	0.6045 ± 0.0633
**CARE (proposed)**	**12**.**42 ± 2**.**36**	**0**.**9001 ± 0**.**0329**	**0**.**6713 ± 0**.**0506**	**0**.**7679 ± 0**.**0370**	**0**.**6157 ± 0**.**0431**

As can be seen from Tables 4 and 5, the Cell Profiler segmentation is characterized by a low *recall* (PHAL: 0.7997, DAPI: 0.8053) and this lead to a lowering of the average *F1*_*SCORE*_ (PHAL: 0.7997, DAPI: 0.8053). Moreover, the mean *jaccard*_*INDEX*_ (PHAL: 0.7939, DAPI: 0.5031) is lower than the proposed one for more than 10%.

Fiji segmentation performance is quite similar to CARE results. The average *F1*_*SCORE*_ achieved with Fiji is slightly lower than those obtained with CARE (PHAL: 0.9249, DAPI: 0.7504). This software is semi-automatic and requires user intervention to function properly. For this reason, the average computational time is about 10 times higher than CARE algorithm.

## Discussion

In this work, we presented a fully automated algorithm for human-derived cardiospheres segmentation in fluorescence microscopy images. The cardiosphere is a promising phenotype for regeneration of the failing human myocardium^11,13,15^, and a promising model of cardiac pathologies such as heart failure^14^. To the best of our knowledge, CARE is the first solution for the automatic segmentation of cells inside *in vitro* 3D aggregates.

The proposed algorithm is capable of recognizing cardiosphere membrane and cells inside fluorescence images. The proposed approach is able to automatically detect cell nuclei in a 3D context without any user interaction. The algorithm was tested on twenty 3D-stacks of human-derived cardiospheres, for a total number of 27 cardiospheres and 1160 slides. Two expert biologists manually annotated cardiosphere membranes for all the images of our dataset. To assess the inter-operator variability in nuclei segmentation, the same manual operators also draw each cell boundary on 100 random images.

The comparison between automatic results and manual annotations showed very high performances for the proposed approach. In detail, the CARE algorithm showed excellent performance in membranes segmentation, with an average *F1*_*SCORE*_ of 0.9497 ± 0.0157 and *jaccard*_*INDEX*_ of 0.9079 ± 0.0255. In cell segmentation, the proposed algorithm obtained a mean *F1*_*SCORE*_ and *jaccard*_*INDEX*_ comparable with respect to two expert operators (Table 1). The CARE algorithm also achieved the highest *F1*_*SCORE*_ compared to other softwares (CellProfiler and Fiji) designed for cell segmentation in fluorescence microscopy. Finally, the proposed method obtained the lowest running time and, respect to other automatic and semi-automatic methods, the best *jaccard*_*INDEX*_.

Thanks to the implementation of adaptive thresholds and optimized object separation, the CARE algorithm achieves high accuracy in cell detection. The efficiency of the proposed method is demonstrated by the low computational times: the CARE algorithm takes only 25 seconds to complete membrane and nuclei segmentation in images with hundreds of cells.

Thanks to the fast and robust cell detection provided by CARE, fully automatic systems for morphological/antigenic characterization of cells inside 3D aggregates can be easily developed. In the next future, a novel cells separation approach will be included within the CARE architecture to further reinforce the detection performance of our method. In addition, we will also test the CARE accuracy in cell segmentation in other *in vitro* 3D aggregates/organoids. Finally, the CARE algorithm could be the tool of election for automated, quantitative analyses of markers distribution within the cardiosphere, aiming at discovering predictive associations between cell mechanical cues and dynamic phenotypic changes.

## Methods

### Cardiosphere culture

Primary cardiospheres (CSs) were isolated as previously described^15^ from right atrial appendage biopsies obtained from three donor patients undergoing elective cardiac surgery during clinically indicated procedures, after informed consent, in an institutional review board approved protocol at the “Umberto I” Hospital, “La Sapienza” University of Rome. All experiments were performed in accordance with relevant guidelines and regulations. Briefly, explant cultures were obtained after mechanical fragmentation and enzymatic digestion (trypsin/EDTA 0.05% for 15 minute at room temperature) of myocardial tissue, and plated on fibronectin-coated petri dishes in the following media recipe: Iscove’s modified Dulbecco’s medium (IMDM) (Sigma-Aldrich) supplemented with 20% FBS (Sigma-Aldrich), 1% penicillin-streptomycin (Sigma-Aldrich), 1% L-glutamine (Lonza, Basel, Switzerland), and 0.1mM2-mercaptoethanol (Gibco, Thermo Fisher Scientific, Waltham, MA, USA). After 4 weeks, explant cells spontaneously migrated from tissue fragments were harvested with EDTA wash and mild trypsinization (trypsin/EDTA 0.05% for 2–3 minute at room temperature). Cells were then plated on poly-D-lysine (BD-Biosciences) coated wells (9000 cells/cm^2^) in the following media: 35% IMDM/65% DMEM/F-12 Mix (Gibco and Lonza), 3.5% FBS, 1% penicillin-streptomycin, 1% L-glutamine, 0.1 mM 2-mercaptoethanol, 1 unit/ml thrombin (Sigma-Aldrich), 1:50 B-27 (Invitrogen), 80 ng/ml bFGF, 25 ng/ml EGF, and 4 ng/ml cardiotrophin-1 (all Peprotech). CSs were harvested by pipetting and centrifugation at 50rcf after 1 week and plated in fibronectin-coated 8-well chamber-slides (Eppendorf) for 3–4 hours to allow attachment. CSs were then fixed with 4% paraformaldehyde for 10 minutes at room temperature, and then subjected to immunofluorescence staining protocols.

### Image database

Twenty 3D-stacks of human-derived cardiospheres obtained from different patients, for a total number of 27 cardiospheres and 1160 slides, were analyzed. Each 3D-stack was acquired using two different lasers to highlight cell membranes (PHAL) and nuclei (DAPI). The voxel size (XYZ) was 0.345 × 0.345 × 0.432 µm/pixel^3^. Each slice had a dimension of 1024 × 1024 pixels (resolution: 0.345 µm/pixel).

For each sample, the number of slices was adapted to include the entire cardiosphere within the Z-stack (average number of slices: 112). Two expert biologists (more than 10 years of experience) manually annotated membranes and nuclei boundaries. The image dataset and the CARE source code are available at https://data.mendeley.com/datasets/tntrkg27st/1.

### CARE algorithm architecture

**Figure 4:**
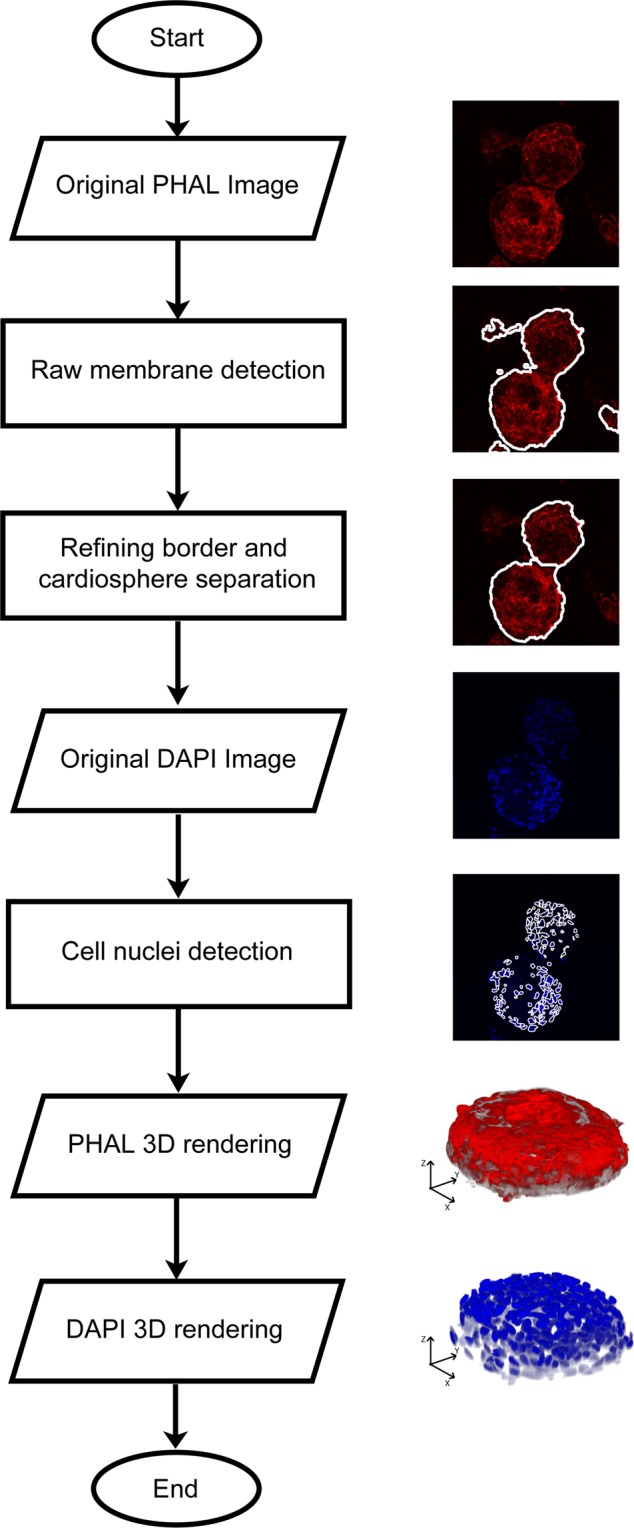
Schematic representation of the CARE algorithm.

The CARE algorithm is designed to automatically segment cardiosphere-derived cells in fluorescence microscopy images. The algorithm is developed in MATLAB (MathWorks, Natick, MA, USA) environment. Image processing and analysis was carried out on a workstation with a 3.1 GHz quad-core CPU and 32-GB of RAM. The procedure of the proposed method is schematically described in Fig. 4. Three main steps compose the processing: PHAL processing, DAPI processing and 3D rendering. In the following sections, a detailed description of the algorithm is provided.

### PHAL processing

The first step of the CARE algorithm is the identification of the cardiosphere membranes by analyzing the 3D stack of the PHAL layer. Then, the identification of the external borders of the cardiospheres is performed by applying an object-based detection scheme to each image of the stack. The core technology of this step is an original object-based detection strategy that we previously developed and adapted to these images^26^, which is briefly described in the following.

In order to analyze the phalloidin, the red layer of the RGB image is extracted and its grayscale histogram is calculated. Then, the Progressive Weighted Mean (*PWM*_*CURVE*_) of the grayscale histogram is computed. Considering a generic class *P* of the histogram (*0* ≤ *P* ≤ *255*), the value of *PWM*_*CURVE*_ for that class is defined as:

5 $$PW{M}_{CURVE}=\frac{{\sum }_{i=0}^{P}{w}_{i}{x}_{i}}{{\sum }_{i=0}^{P}{w}_{i}}$$

where, *w*_*i*_ is the histogram count for the *i*^*th*^ class and *x*_*i*_ is the respective bin location. The *PWM*_*CURVE*_ is evaluated for each class of the histogram as the weighted mean of all the grayscale histogram values up to that class. As the trend of *PWM*_*CURVE*_ depends on the shape of the histogram, relevant features based on image color distribution can be extracted using this function. In particular, inflection points of *PWM*_*CURVE*_ may be potential threshold values for performing cardiospheres segmentation, as they represent local stability points of the grayscale histogram. In particular, cardiosphere membranes can be defined as objects with an intensity higher than a threshold value that can be unambiguously identified as follows. First of all, the *PWM*_*CURVE*_ is fitted with a 10^th^ order polynomial function with the aim to estimate its inflection points (*candidate thresholds*). Then, the grayscale image is segmented using all the candidate thresholds and the standard deviation of detected objects intensity is evaluated for all thresholds. Among candidate thresholds, the algorithm considers as *initial threshold* value the one that identifies objects with the lowest standard deviation. This condition on the standard deviation is imposed to obtain homogeneous objects.

**Figure 5:**
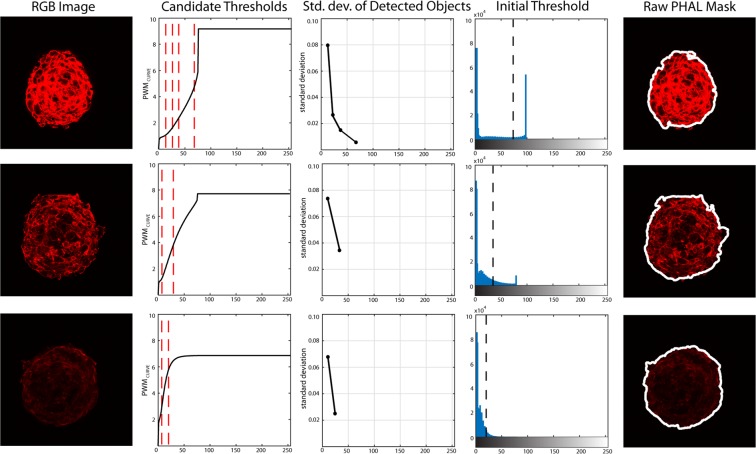
Processing for obtaining the initial threshold for different images of the stack with different high variation of intensity (PHAL layer). Starting from the red layer of the RGB image, the *PWM*_*CURVE*_ is estimated from its grayscale histogram. Then, candidate thresholds are evaluated as inflection points of the curve (red dotted lines). The standard deviation of detected objects intensity using candidate thresholds is calculated and the initial threshold is determined as the one with the lowest standard deviation. In the last column, the application of the initial threshold on the RGB image is shown.

The processing steps for obtaining the initial threshold are illustrated in Fig. 5, where images with three different laser intensities are presented as explanatory examples. From the results presented in Fig. 5, it can be appreciated the robustness of the proposed method for cardiospheres border identification, where an optimal threshold image intensity value is selected, regardless of the shape both of the image histogram and of the cardiosphere.

The herein develop method also includes an automatic strategy for the refinement of the shapes of the objects detected. Preliminarily, detected objects with area less than 1200 µm^2^ are deleted because they are too small to be considered as cardiospheres. Then, starting from the first frame of the stack, the CARE algorithm also performs an iterative four-steps processing procedure to further clean the obtained masks:

1. definition of *reference frame* as the current frame;
2. definition of *realign frame* as the next frame after the *reference frame*;
3. deletion of all the objects inside the *realign frame* with an overlapping lower than 75% with *reference frame* objects;
4. move on to next frame (with the current realign frame becoming the next current frame).

**Figure 6:**
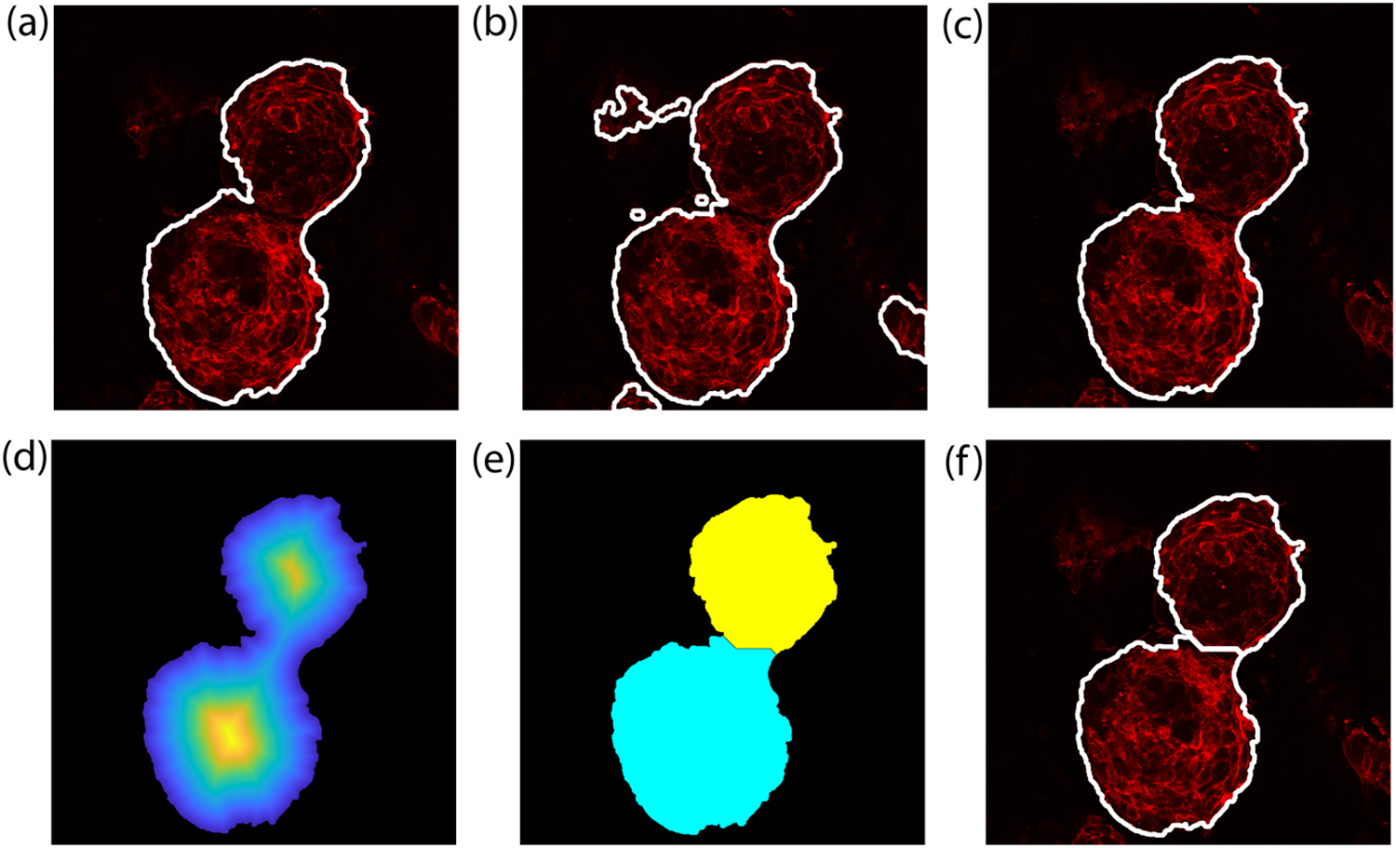
Schematic representation of the CARE algorithm. Processing of cardiospheres membranes. First row shows the refining operation of cardiospheres external edges while second row illustrates the separation process of touching cardiospheres. (**a**) Current frame (reference frame), (**b**) realign frame (next frame), (**c**) realign frame after refining, (**d**) distance transform of the membrane mask, (**e**) application of the marker-based watershed for cardiosphere separation, (**f**) final membrane mask on RGB image.

The procedure described above is extended to all the images of the stack. With this operation, all previously segmented objects that do not belong to the cardiosphere are deleted. An example of the refining process is presented in Fig. 6a–c.

Our object-based detection technology has a high sensitivity, but sometimes it may lead to suboptimal profiles, possibly given by two or more cardiospheres that are very close to each other. In such a case, the automatic algorithm may depict them as a single object. However, our technique incorporates a post-processing step to overcome this issue. A marked-based watershed^26,27^, was implemented to separate “fused” cardiospheres. To identify marker positions, the distance transform of the membrane binary mask is calculated, and the local maxima are identified using the extended-maxima transform^27^. Technically, the extended-maxima transform estimates regional maxima by searching in N-connected neighborhoods. For this application, a neighborhood size of N = 20 pixels (equal to 6.91 µm) was empirically set, based on the observation that it guarantees an effective and affordable output in terms of cardiospheres separation. The separation process of “fused” cardiospheres is illustrated in Fig. 6.

### DAPI processing

**Figure 7:**
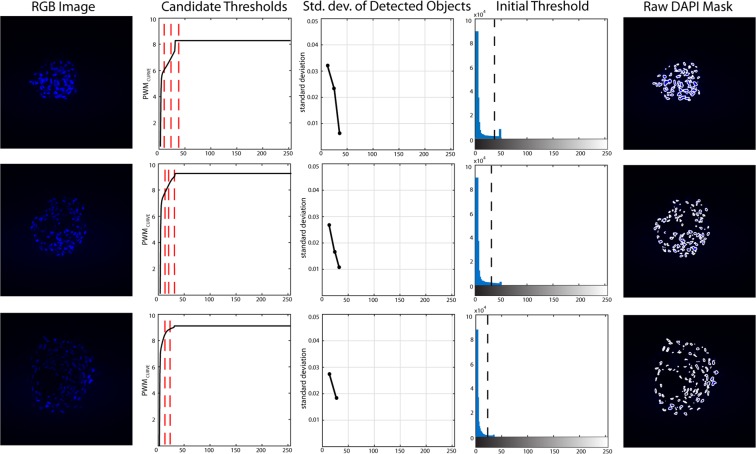
Processing for obtaining the initial threshold for different images of the stack with different high variation of intensity (DAPI layer). Starting from the blue layer of the RGB image, the *PWM*_*CURVE*_ is estimated from its grayscale histogram. Then, candidate thresholds are evaluated as inflection points of the curve (red dotted lines). The standard deviation of detected objects intensity using candidate thresholds is calculated and the initial threshold is determined as the one with the lowest standard deviation. In the last column, the application of the initial threshold on the RGB image is shown.

**Figure 8:**
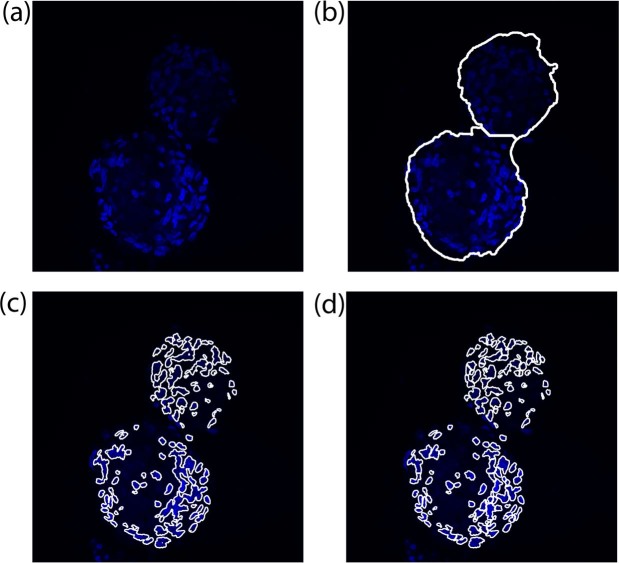
Processing of DAPI layer. (**a**) Original RGB image, (**b**) membrane mask applied to the image, (**c**) raw nuclei detection, (**d**) cell nuclei separation using a marker-based watershed.

After cardiospheres border segmentation, the proposed method analyzes the 3D stack of the DAPI layer (Fig. 7). Starting from the original RGB image (Fig. 8a), the cardiospheres segmentation is applied to each frame (Fig. 8b). All objects outside the mask are excluded from the analysis, as they do not belong to the cardiosphere. The same object-based detection used for the PHAL processing is applied for cell nuclei segmentation to obtain a raw mask of cells inside each cardiosphere (Fig. 8c). In the acquired images, cell nuclei are very often close to each other and the algorithm connects them as a single structure^28^. For this reason, also at this stage of the investigation a marker-based watershed is applied in order to separate fused nuclei (Fig. 8d).

### 3D rendering

**Figure 9:**
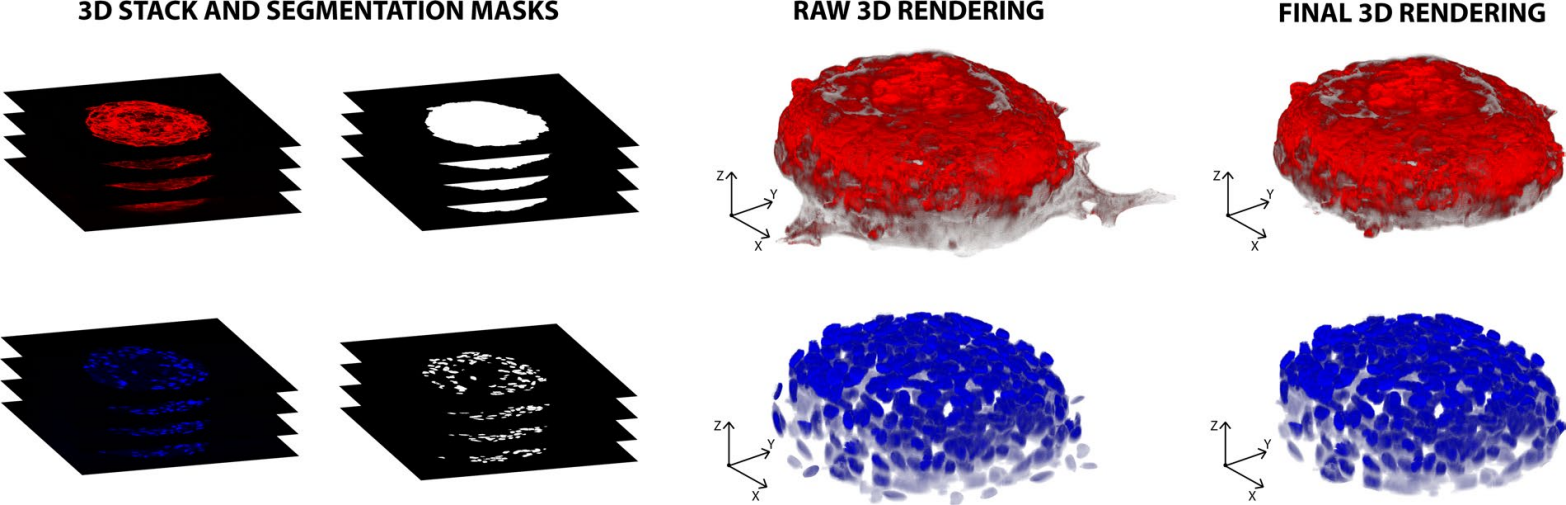
Cardiosphere 3D rendering before and after the cut-off frame estimation. PHAL and DAPI mask are combined to obtain the raw volumes. Then, the proposed algorithm identifies the frame in which there is the first contact between the cardiosphere and the surface. The final 3D rendering is achieved by excluding all the slides after the cut-off frame.

The 3D rendering of cardiospheres is obtained combining the segmentation masks obtained as mentioned above with the corresponding RGB image (Fig. 9). Unfortunately, this operation is not sufficient to ensure a proper 3D reconstruction of the volume of cardiospheres, because it is affected by the border effect in the region where the cardiosphere is in contact with the surface on which it grows. To overcome this limitation, the method proposed here identifies the frame where the first contact between the cardiosphere and the support surface occurs (*cut-off frame*). To do that, starting from the first image of the stack, three conditions are checked on the border segmentation mask. If at least one of these conditions is satisfied, the slide is labeled as *cut-off frame* and the remaining images are not used for 3D rendering:

1. Grayscale intensity - if in the image *i-th* of the stack the grayscale average intensity inside the segmented border mask is lower than 0.20, then the image is too dark to be considered for 3D rendering;
2. Shape difference - if the area difference between the segmented border in frame *i-1* and frame *i* is greater than 30%, then the cardiosphere is starting to spread on the surface;
3. Shape solidity - if the segmented border mask solidity is less than 0.60, then the shape is so irregular that it cannot belong to a single cardiosphere. Solidity of a region is defined as the ratio between its area and convex area. Since it is expected that cardiospheres are convex objects, the solidity is used as feature for the identification of the *cut-off frame*.

Figure 9 shows a 3D rendering before and after the estimation of the cut-off frame. Through the process described above, the CARE algorithm produces two renderings: (i) the 3D volume of the external border of the cardiosphere and (ii) the 3D volume of all the cell nuclei inside the cardiosphere.

## Supplementary information

Supplementary information

## Data Availability

The CARE algorithm and the dataset used during the current study are made available as indicated in the Method section.

## References

[1] Woon MT, Kamp TJ (2017). Stem Cells: Put to the test. Elife.

[2] Jones DL, Wagers AJ (2008). No place like home: anatomy and function of the stem cell niche. Nat. Rev. Mol. cell Biol..

[3] Roeder I, Loeffler M, Glauche I (2011). Towards a quantitative understanding of stem cell–niche interaction: Experiments, models, and technologies. Blood Cells, Mol. Dis..

[4] Roeder I, Lorenz R (2006). Asymmetry of stem cell fate and the potential impact of the niche. Stem Cell Rev..

[5] Murrow LM, Weber RJ, Gartner ZJ (2017). Dissecting the stem cell niche with organoid models: an engineering-based approach. Development.

[6] Kirouac DC, Zandstra PW (2008). The systematic production of cells for cell therapies. Cell Stem Cell.

[7] Shakiba N, Zandstra PW (2017). Engineering cell fitness: lessons for regenerative medicine. Curr. Opin. Biotechnol..

[8] Blagoev KB (2011). Organ aging and susceptibility to cancer may be related to the geometry of the stem cell niche. Proc. Natl. Acad. Sci..

[9] Yachie‐Kinoshita A (2018). Modeling signaling‐dependent pluripotency with Boolean logic to predict cell fate transitions. Mol. Syst. Biol..

[10] Tewary, M. *et al*. A stepwise model of Reaction-Diffusion and Positional-Information governs self-organized human peri-gastrulation-like patterning. *Development* dev-149658 (2017). 10.1242/dev.149658 PMC5769627 28870989

[11] Gaetani R (2014). Different types of cultured human adult cardiac progenitor cells have a high degree of transcriptome similarity. J. Cell. Mol. Med..

[12] Gaetani R (2012). Cardiac tissue engineering using tissue printing technology and human cardiac progenitor cells. Biomaterials.

[13] Barile L (2007). Cardiac stem cells: isolation, expansion and experimental use for myocardial regeneration. Nat. Rev. Cardiol..

[14] Pesce M, Messina E, Chimenti I, Beltrami AP (2017). Cardiac mechanoperception: a life-long story from early beats to aging and failure. Stem Cells Dev..

[15] Chimenti I (2017). Stem cell spheroids and *ex vivo* niche modeling: rationalization and scaling-up. J. Cardiovasc. Transl. Res..

[16] Messina E (2004). Isolation and expansion of adult cardiac stem cells from human and murine heart. Circ. Res..

[17] Desmaison A (2018). Impact of physical confinement on nuclei geometry and cell division dynamics in 3D spheroids. Sci. Rep..

[18] Niraimathi, M. M. F. A. & Seenivasagam, V. A Marker Controlled Watershed Algorithm with Priori Shape Information for Segmentation of Clustered Nuclei. *Int*. *J*. *Adv*. *Res*. *Comput*. *Sci*. **2** (2011).

[19] Coelho, L. P., Shariff, A. & Murphy, R. F. Nuclear segmentation in microscope cell images: a hand-segmented dataset and comparison of algorithms. in *Biomedical Imaging: From Nano to Macro*, *2009*. *ISBI’09*. *IEEE International Symposium on* 518–521 (IEEE 2009). 10.1109/ISBI.2009.5193098 PMC2901896 20628545

[20] Li G (2008). Segmentation of touching cell nuclei using gradient flow tracking. J. Microsc..

[21] Sokolova M, Lapalme G (2009). A systematic analysis of performance measures for classification tasks. Inf. Process. Manag..

[22] Real R, Vargas JM (1996). The Probabilistic Basis of Jaccard’s Index of Similarity. Syst. Biol..

[23] Breslow N (1970). A generalized Kruskal-Wallis test for comparing K samples subject to unequal patterns of censorship. Biometrika.

[24] Carpenter AE (2006). CellProfiler: image analysis software for identifying and quantifying cell phenotypes. Genome Biol..

[25] Schindelin J (2012). Fiji: an open-source platform for biological-image analysis. Nat. Methods.

[26] Salvi M, Molinari F (2018). Multi-tissue and multi-scale approach for nuclei segmentation in H&E stained images. Biomed. Eng. Online.

[27] Xu H, Lu C, Mandal M (2014). An Efficient Technique for Nuclei Segmentation Based on Ellipse Descriptor Analysis and Improved Seed Detection Algorithm. IEEE J. Biomed. Heal. Informatics.

[28] Patwardhan A (2017). Cutting edge: building bridges between cellular and molecular structural biology. Elife.

