# Supplementary material for: Automated Segmentation of Fluorescence Microscopy Images for 3D Cell Detection in human-derived Cardiospheres
Source: Sci Rep. 2019 Apr 30;9:6644. doi: 10.1038/s41598-019-43137-2 (PMC6491482; doi:10.1038/s41598-019-43137-2)
Supplement: Supplementary file 1 — Supplementary information [file 41598_2019_43137_MOESM1_ESM.pdf]

Automated Segmentation of Fluorescence Microscopy Images for 3D Cell Detection in human-derived Cardiospheres

Massimo Salvi[1,*], Umberto Morbiducci[2], Francesco Amedeo[3], Rosaria Santoro[3], Francesco Angelini[4], Isotta Chimenti[4], Diana Massai[2], Elisa Messina[4], Alessandro Giacomello[4], Maurizio Pesce[3], and Filippo Molinari[1]

[1]Department of Electronics and Telecommunications, Politecnico di Torino, Turin, 10129, Italy

[2]Department of Mechanical and Aerospace Engineering, Politecnico di Torino, Turin, 10129, Italy

[3]Unità di Ingegneria Tissutale Cardiovascolare, Centro Cardiologico Monzino, IRCSS, Milan, 20138, Italy

[4]Department of Medical-Surgical-Sciences and Biotechnology, Sapienza University of Rome, Rome, 00185, Italy

* corresponding author massimo.salvi@polito.it

Supplementary Information

CellProfiler pipeline

The pipeline implemented in CellProfiler is the same one used for cell segmentation in a previous work (Buggenthin et al, *An automatic method for robust and fast cell detection in bright field images from high-throughput microscopy*, BMC bioinformatics, 2013, 14.1: 297). The following modules were sequentially called for each image: Correct Illumination (Gaussian filter, Average object size: 60 px), Apply threshold (Otsu global), Identify primary objects (Typical diameter of cells: 5 to 50, splitting method: Intensity, method to draw dividing lines: Shape), Convert objects to image (saved binary mask).

Fiji algorithm

The semi-automatic pipeline implemented in Fiji consisted of: (i) conversion of RGB image into grayscale, (ii) manual intensity thresholding, (iii) hole filling and (iv) small particles removal (all objects with area lower than 10 μm^2 are deleted). For the nuclei segmentation, here an additional step was included in the analysis: (v) automatic cell separation based on watershed transform (Beucher and Mayer, *The morphological approach to segmentation: the watershed transformation*, Optical Engineering-New York-Marcel Dekker Incorporated, 1992, 34: 433-433).
